# Neurasthenia in Children

**Published:** 1903-10

**Authors:** 


### NEURASTHENIA IN CHILDREN.

DR. J. M. TAYLOR says the acquired form of neurasthenia, so common in adults, secondary to a large variety of exhausting conditions, is rare in children, but occurs with sufficient frequency to warrant attention. There are also many cases of feebleness or frailty, which, in essence, are instances of "nerve tire" and are the product of injudicious or unfortunate environment or upbringing so closely allied to hereditary causes that they cannot well be separated. Many of these cases will continue through life; a much larger number of them are capable of most satisfactory improvement. The manifestations of neurasthenia in children are obvious enough, if rightly interpreted. They may take the form of undue fondness for one pursuit, such as reading, or a hypersensitiveness to external impressions, or an overstrenuousness in play or work, a hypertension, or in other instances which are to be carefully discriminated from the last class, a hypotension.

The treatment of this condition requires the utmost care to all details of air, diet, exercise, etc. A modified form of rest cure is given in detail.—*Post-Graduate.*

